# Death and survival in *Streptococcus mutans*: differing outcomes of a quorum-sensing signaling peptide

**DOI:** 10.3389/fmicb.2015.01176

**Published:** 2015-10-23

**Authors:** Vincent Leung, Delphine Dufour, Céline M. Lévesque

**Affiliations:** Dental Research Institute, Faculty of Dentistry, University of Toronto, Toronto, ON, Canada

**Keywords:** quorum-sensing, peptide pheromone, persister cells, bacterial suicide, *Streptococcus mutans*, stress response, phenotypic heterogeneity

## Abstract

Bacteria are considered “social” organisms able to communicate with one another using small hormone-like molecules (pheromones) in a process called quorum-sensing (QS). These signaling molecules increase in concentration as a function of bacterial cell density. For most human pathogens, QS is critical for virulence and biofilm formation, and the opportunity to interfere with bacterial QS could provide a sophisticated means for manipulating the composition of pathogenic biofilms, and possibly eradicating the infection. *Streptococcus mutans* is a well-characterized resident of the dental plaque biofilm, and is the major pathogen of dental caries (cavities). In *S. mutans*, its CSP QS signaling peptide does not act as a classical QS signal by accumulating passively in proportion to cell density. In fact, particular stresses such as those encountered in the oral cavity, induce the production of the CSP pheromone, suggesting that the pheromone most probably functions as a stress-inducible alarmone by triggering the signaling to the bacterial population to initiate an adaptive response that results in different phenotypic outcomes. This mini-review discusses two different CSP-induced phenotypes, bacterial “suicide” and dormancy, and the underlying mechanisms by which *S. mutans* utilizes the same QS signaling peptide to regulate two opposite phenotypes.

## Introduction

Oral streptococci are pioneer colonizers of the oral cavity and are abundant in the dental plaque, a tooth-associated biofilm (Lazarevic et al., 2009). *Streptococcus mutans* is an important constituent of the dental plaque biofilm (Kolenbrander et al., 2006). It is widely recognized as a key etiological agent of dental caries (cavities; Loesche, 1986), and is a member of the predominant microflora of caries lesions due in part to the production of organic acids from dietary sugars and its resistance to the resulting low pH caused by acid accumulation (Takahashi and Nyvad, 2008). *S. mutans* also possesses the ability to combat harsh physiological conditions of the oral environment using its multiples two-component systems (Lemos and Burne, 2008; Smith and Spatafora, 2012). Recent studies have shown that *S. mutans* uses a canonical Gram-positive two-component quorum-sensing (QS) system to regulate expression of genes controlling multiple phenotypes. In this mini-review, we will discuss how *S. mutans* uses the same QS signal molecule to control its death by “suicide” and its survival through the production of dormant persister cells.

## Quorum-sensing and Stress Response

Quorum-sensing is a cell-to-cell communication system used by bacteria to coordinate bacterial behavior at the whole population level through the use of small diffusible signaling molecules (Ng and Bassler, 2009). These signaling molecules accumulate in the environment in a cell density-dependent manner. Once a threshold concentration is reached (“quorum” or the number of bacteria required to activate the QS system), the QS molecule initiates a signaling cascade culminating in a population-wide differential regulation of target genes enabling bacteria to act as multicellular organisms. Using QS, bacteria regulate gene expression in response to the concentration of signaling molecules produced and released into the local environment by itself or the same species (intraspecies QS) or other bacteria of different species (interspecies QS). A wide array of bacterial phenotypes are controlled by QS including biofilm development, bioluminescence, sporulation, motility, conjugation, genetic competence, and bacteriocin production (Ng and Bassler, 2009). Many opportunistic pathogens also rely on QS circuits as central regulators of virulence expression (Rutherford and Bassler, 2012).

In streptococci, the best characterized QS system, called CSP-ComDE, is achieved by the production and detection of signaling molecules in the form of a small peptide named CSP (competence-stimulating peptide) pheromone. The development of genetic competence, the physiological state in which cells are able to take up exogenous DNA, was the first phenotype found to be regulated by the CSP-ComDE QS in streptococci (reviewed, in Oggioni and Morrison, 2008). Since then, an interesting alternative view related to the function of the CSP-ComDE QS system was proposed. It was suggested that the development of genetic competence was triggered as a general stress response. In this case, stress signals (e.g., nutrient starvation, DNA damage, etc.) could activate the competence pathway to promote recombinational mechanisms for repair of damaged DNA or other functions for repairing cellular damages (Claverys et al., 2006). Global transcriptome analyses of CSP-regulated genes in *S. mutans* and *S. pneumoniae* presented evidence that the CSP pheromone used to regulate expression of the competence regulon related to genetic exchange, was also activating expression of stress-response genes (Claverys et al., 2006; Perry et al., 2009b). Furthermore, *S. mutans* has been shown to initiate a response to the onset of various adverse environmental stressors, including heat shock, oxidative stress, acidic pH, amino acid starvation, and even antibiotic treatment, by actively producing the CSP pheromone (Perry et al., 2009b; Leung and Lévesque, 2012). Altogether, these studies suggested that CSP most probably functions as a stress-inducible pheromone or “alarmone” by triggering the signaling to the bacterial population to initiate an adaptive response that results in different phenotypic outcomes.

## CSP-ComDE and ComRS QS Systems in *S. mutans*

**FIGURE 1:**
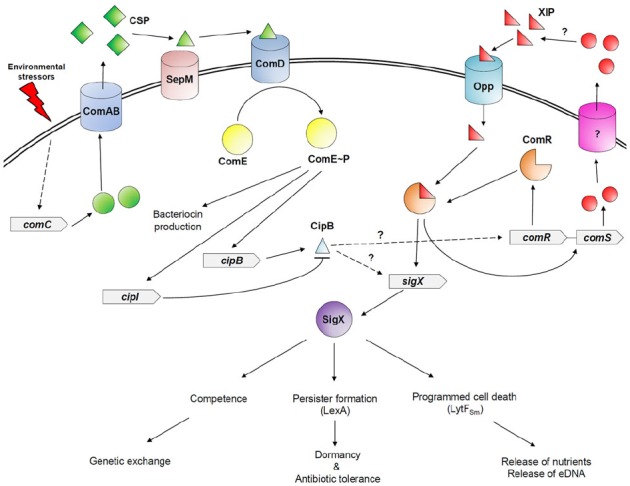
**The CSP-ComDE and ComRS quorum-sensing systems in ***S. mutans***.** Particular environmental stressors, such as those encountered in the oral cavity, induce the expression of the CSP-encoding gene (*comC*). The CSP propeptide is processed and exported by the specific ABC transporter, ComAB, into the extracellular milieu. A final post-export processing step is then mediated by the SepM protease. When the mature CSP pheromone accumulates to surpass a threshold concentration, it directly binds to the membrane-bound ComD receptor triggering its autophosphorylation, and the subsequent activation of the cognate cytoplasmic response regulator ComE by phosphorylation. Activated ComE directly activates the expression of bacteriocin-related genes, including *cipB* and *cipI*. The ComRS quorum-sensing system is composed of a double-tryptophan-containing signal peptide XIP encoded by *comS* gene, and its transcriptional regulator ComR. Contrary to the CSP-ComDE quorum-sensing system in which the CSP pheromone is sensed outside the cell, the ComRS system involves sensing of XIP inside the cell after its internalization through an Opp transporter in a peptide-free medium. The XIP/ComR complex activates transcription of *comR* and *comS* genes creating a positive feedback loop, and ComR induces the expression of *sigX*. Although CipB activates expression of SigX-dependent genes such as *lytF_Sm_* involved in CSP-induced PCD, it is unclear how both quorum-sensing systems are linked in a peptide-rich medium. It was recently suggested that CipB could also permeabilize the cell after its insertion into the membrane. The permeabilization of the cell membrane would enable the import of XIP, which, in association with ComR regulator, would directly activate SigX responsible for the development of genetic competence, stress-induced persisters, and CSP-induced PCD.

The CSP-ComDE system is composed of the secreted linear CSP pheromone, and the ComDE two-component system (Li et al., 2001). The intracellularly generated CSP propeptide contains a conserved Gly-Gly motif, which is recognized and cleaved off during its export outside the cell by the specific ABC transporter, ComAB (Petersen and Scheie, 2000). A final post-export processing step is mediated by the extracellular SepM membrane-bound protease to generate a derivative that lacks the three C-terminal amino acid residues (Petersen et al., 2006; Hossain and Biswas, 2012). This truncated derivative is the functional form of CSP, and called CSP pheromone. The CSP pheromone accumulates in the milieu and upon reaching a specific threshold concentration, it directly interacts with the membrane-bound histidine kinase receptor, ComD, to orchestrate a signaling response. The interaction of CSP pheromone with ComD triggers the dimerization and autophosphorylation of the receptor, and initiates the phosphorylation and subsequent activation of the cytoplasmic response regulator ComE (Hung et al., 2012). Activated ComE directly activates the expression of several genes encoding bacteriocins and bacteriocin-like peptides (Kreth et al., 2006; Perry et al., 2009b), and indirectly regulates SigX, the alternative sigma factor, involved in the control of the competence regulon (Aspiras et al., 2004; Figure 1).

A second type of intraspecies QS system, named ComRS, has been recently described in *S. mutans* (Mashburn-Warren et al., 2010; Fontaine et al., 2013). This system is composed of the small XIP (SigX-induced peptide) molecule and its transcriptional regulator ComR. Recently, it has been demonstrated that the ComRS system was the proximal regulator of *sigX* (Mashburn-Warren et al., 2010). Interestingly, SigX activity is highly sensitive to the growth medium used to cultivate the cells. CSP pheromone activates SigX in a nutrient-rich medium, while XIP stimulates SigX only in a chemically-defined medium devoid of exogenous peptides (Son et al., 2012). A recent study showed that SigX directly controls expression of ComE regulator but only when cells are cultivated in a peptide-free chemically-defined medium (Reck et al., 2015).

Although the interconnection between the CSP and XIP signaling pathways remains not completely understood, it was demonstrated that the CSP-inducible *cipB* gene directly regulated by ComE was important for the activation of SigX in a nutrient-rich medium (Dufour et al., 2011). The presence of *cipB* gene is highly variable in strains and isolates of *S. mutans* (Kamiya et al., 2008). A correlation was found in *S. mutans* clinical isolates that were capable of undergoing natural competence and the presence of *comCDE*, *cipB*, and *sigX* genes (Palmer et al., 2013). In strains that lack a functional CipB or are unable to undergo natural competence, it is speculated that the main function of CSP-ComDE is to regulate the production of bacteriocins.

## Bacterial “Suicide” or Altruistic Programmed Cell Death

Programmed cell death (PCD) is a genetically-encoded process responsible for triggering cellular self-destruction. In higher organisms, PCD is known as apoptosis, and is an essential mechanism responsible for cellular development, organ/tissue differentiation, homeostasis, and eradication of defective and/or damaged cells (Ameisen, 1996; Jacobson et al., 1997). By contrast, the beneficial effects of PCD or “suicide” for a single-celled organism remains unclear. However, the fact that most bacteria do not live solitary lives but live in biofilms, suggest that bacterial biofilms most likely act as multicellular organisms (López et al., 2010). The concept of bacterial suicide becomes then obvious when examining biofilms. As such, the biofilm lifestyle may allow altruistic behaviors that can contribute to the continued survival of siblings in a stressed population. Bacterial PCD has been observed when these populations encounter stress conditions such as amino acid starvation, pH changes, oxygen radicals, high temperature, DNA damage, phage attack, and antibiotics (Lewis, 2000; Engelberg-Kulka et al., 2006; Tanouchi et al., 2013). In fact, bacterial PCD is a kind of altruistic act that provides a way for the species to survive stresses at the expense of some of its cells. For example, the death by suicide of a subpopulation may leave more resources in the milieu or the surviving population can even grow on constituents leaking out of dead siblings, and thereby improving long-term survival of the species. Targeted suicide of a subpopulation of cells within a biofilm may also promote the formation of “voids” or channels that facilitate the transport of water and nutrients (Mai-Prochnow et al., 2004). The death by suicide of a fraction of the population under particular conditions thus represents an ecological advantage where some bacteria die to benefit the whole population.

In *S. mutans*, a PCD pathway regulated by CSP-ComDE has been discovered and characterized by some members of our group. As part of its ability to adapt to stress, *S. mutans* activates production of its CSP pheromone. Under high levels of CSP, a small fraction of the population (<10%) undergoes death by suicide (Qi et al., 2005; Perry et al., 2009b). CipB was identified as a major effector in the CSP-induced PCD process. It was discovered that cellular self-destruction of a small fraction of the population was due to intracellular accumulation of CipB in the producing cells (Perry et al., 2009b). CSP-induced suicide can be prevented by the action of the immunity factor CipI most probably through CipB sequestration. Interestingly, CipB participates in CSP-induced suicide at the transcriptional level by indirectly regulating SigX-dependent genes such as *lytF_Sm_* encoding a conserved murein hydrolase (Dufour et al., 2011; Dufour and Lévesque, 2013). Cell death occurs via autolysis through the hydrolase activity of the enzyme on the producer cells. LytF_*Sm*_ is also an important actor involved in CSP-induced PCD since no lysis of the cells can be observed in a Δ*lytF_Sm_* mutant. The *lytF_Sm_* gene was also found upregulated when cells were challenged by environmental stressors encountered in the oral cavity reinforcing its role in CSP-induced PCD (Dufour and Lévesque, 2013). It was recently suggested that CipB could also permeabilize the cell after its insertion into the membrane. The permeabilization of the cell membrane would enable the import of XIP, which, in association with ComR regulator, would directly activate SigX (Reck et al., 2015).

Evidence was presented that CSP-induced PCD was involved in the release of extracellular DNA in the biofilm, which contributes to the architecture of the biofilm matrix and may provide a mechanism for the dissemination of fitness-enhancing genes under stress (Perry et al., 2009a). Recently, it was demonstrated that the viability of a Δ*lytF_Sm_* mutant in a long-term survival assay was significantly lower than that observed for the parent strain, suggesting that surviving cells may benefit from the nutrients released through the action of the autolysin activated by the CSP-induced PCD pathway (Dufour and Lévesque, 2013).

## Survival Through Dormancy

Within a given population of bacteria, a small subpopulation of cells enters into a state of dormancy. These non-growing dormant cells, called persisters, are tolerant to all antibiotics currently in use without expressing a drug resistance mechanism. Persisters are not mutants but rather phenotypic variants of the wild-type strain that arise in a clonal population of genetically identical cells (Lewis, 2010). By entering into a growth-arrested physiological state, persisters are shutting down the activity of essential cellular processes targeted by antibiotics allowing them to survive. It has been discovered that persisters play a significant role in the high-level drug tolerance of biofilms as well as contributing toward chronic biofilm infections (Fauvart et al., 2011). Most studies have described persister formation as a “spontaneous” mechanism (Cohen et al., 2013). In fact, the existence of a small sub-population of persisters occurring spontaneously in any growing bacterial population reflects a population-level strategy of survival in a rapidly changing environment (“bet-hedging” strategy). Recent studies revealed that the formation of persisters can also be governed by deterministic mechanisms following exposure to stress or induced by QS molecules, including phenazine pyocyanin and acyl-homoserine lactone in *Pseudomonas aeruginosa*, indole in *E. coli* and *Salmonella typhimurium*, and CSP pheromone in *S. mutans* (reviewed, in Maisonneuve and Gerdes, 2014).

Since *S. mutans* is using its CSP pheromone as a stress inducible alarmone, it is not surprising that the CSP-ComDE QS system is used by *S. mutans* as a deterministic mechanism for persister formation. It was demonstrated that different stresses encountered in the oral cavity increased the level of persisters. However, this stress inducible persister phenotype was abolished in any QS-deficient mutants unable to produce, detect, or respond to the CSP pheromone (Leung and Lévesque, 2012). More recently, it was shown that an intact signal relay between ComDE, ComRS to the activation of SigX was required for the stress-induced persistence phenotype (Leung et al., 2015). Recently, it was revealed that the LexA transcriptional regulator enacts a significant regulatory role in the formation of the CSP-induced persisters as this inducible persistence phenotype was abolished in a Δ*lexA* mutant. Transcriptome analyses identified specific genes regulated by LexA during the exposure to the CSP pheromone, specifically those involved in sugar and amino acid metabolism, CRISPR (clustered regularly interspaced short palindromic repeat) system, and an autolysin, all of which appeared to contribute toward the inducible persistence phenotype. Thus, these results showed the importance of QS signaling in mounting an adaptive stress response within a subpopulation of *S. mutans* to form dormant persisters for survival of the species.

## Death and Survival, a Result of Phenotypic Heterogeneity and Bistability

Bacterial populations can be genetically homogenous. However, even within isogenic populations, cells can display various phenotypes (Kussell and Leibler, 2005). For example, a clonal population may have two (bistability) or multiple (multistability) subpopulations with distinct phenotypes. Phenotypic variability can occur spontaneously (stochastic switching) or in response to environmental perturbations (responsive switching). Stochastic variation is due to “noise” in gene expression patterns, and is a key determinant of phenotypic variation (Venning et al., 2008). In this case, cellular fates result from amplified noise in gene expression, where slight differences in mRNA or protein levels of a particular gene will generate phenotypic heterogeneity. There are two types of fluctuations that can occur during gene expression. Intrinsic noise is the randomness inherent to the biochemical process of gene expression itself (transcription, translation), and extrinsic noise, where fluctuations in other factors influence gene expression (the number of RNA polymerases or ribosomes per cell; Raj and van Oudenaarden, 2008). Furthermore, these noises can be exacerbated from specific regulatory feedback systems and maintain phenotypic switching into bistable or multistable subpopulations.

In the context of *S. mutans* CSP-induced PCD, much of the phenotypic outcome is influenced by the ability of the cells to respond to the CSP pheromone efficiently. In *S. pneumoniae*, CSP induces an unimodal response under laboratory conditions, where approximately 100% of cells in the population become naturally competent (Martin et al., 2010). There is no bifurcation or bimodality in the population, and this was found to be due to the transcriptional read-through of the tRNA^ARG5^ gene upstream of *comCDE* locus encoding the CSP-ComDE QS system in *S. pneumoniae*. The read-through ensures cells exceed the threshold of CSP activation of the *comCDE* regulon. In *S. mutans*, it was found that the CSP pheromone induces a bimodal response, where only a fraction of the population undergoes cell suicide as a result of the CipB-led killing (Perry et al., 2009b). These results were confirmed in a separate study using a combination of flow cytometry sorting of cells guided by a green fluorescent protein (GFP) driven by the *sigX* promoter and DNA microarrays for comparison of transcriptional profiles between the GFP+ and GFP– populations (Lemme et al., 2011). It was found that the GFP– subpopulation had low *comE* and *cipI* expression upon CSP exposure, while GFP+ subpopulation had high *comE* and *sigX* induction. A second bifurcation of this GFP+ subpopulation occurred at the level of *cipI* expression. One fraction highly expressed *cipI* and became competent cells, while the second fraction expressed low *cipI* actively underwent CSP-induced PCD due to high expression of *cipB*. The subpopulation that undergoes cell suicide would potentially contribute extracellular DNA and other secondary signals for uptake by the survivors.

The switch from “normal” growing cells to dormancy and vice versa is stochastic and involves noise in gene expression. Regarding the formation of persisters, it is now believed that the persistence phenotype is the end result of a stochastic switch in the expression of toxin-antitoxin (TA) modules (Jayaraman, 2008). In *S mutans*, fluctuations in the levels of both MazEF and RelBE type II TA modules exerted an increase in persisters (Leung and Lévesque, 2012). In contrast, ectopic expression of Fst-Sm/srSm type I TA module decreased persister formation in suggesting that the decrease could be related to persisters awakening from dormancy (Koyanagi and Lévesque, 2013). It is currently unknown if the CSP pheromone creates an imbalance in the intracellular levels of these TA modules. Although the importance of an intact QS pathway initiating from CSP binding to the ComD receptor to the eventual activation of SigX is necessary for the formation of CSP-induced persisters, the precise mechanism is still unknown and warrants further investigation. Ultimately, it is the bimodality of *sigX* response that gives rise to the differing phenotypic outcomes described in this mini-review.

## Concluding Remarks

It is quite obvious that *S. mutans* utilizes its CSP-ComDE QS system as a means to convey an adaptive stress response that yields phenotypic heterogeneity within a given clonal population. One of the cell subpopulations undergoes suicide as a potentially altruistic act to eliminate damaged individual cells upon the exposure to adverse environmental conditions, and possibly provide nutrients to benefit the bacterial population as a whole. Another cell fraction enters a dormant state that is multidrug tolerant for the overall survival of the species. Phenotypic variation is the result of bistability, and we are only scratching the surface of how these phenotypes arise through the QS system of the bacteria. The research presented in this mini-review definitely acknowledges the importance of QS and its role in cell-to-cell variability for the generation of phenotypic variable populations. Interference with QS signaling constitutes a promising avenue toward development of novel therapeutics for biotechnological and medical applications. For instance, QS peptides could be useful for inducing targeted suicide. On the other hand, quenching QS mechanisms could be considered to prevent deterministic persistence and genetic heterogeneity through the spreading of potential antibiotic resistance genes.

## Author Contributions

Conception/design of the work: VL, DD, CL; drafting the work: VL, DD, CL; final approval of the manuscript to be published: VL, DD, CL; agreement to be accountable for all aspects of the work: VL, DD, CL.

### Conflict of Interest Statement

The authors declare that the research was conducted in the absence of any commercial or financial relationships that could be construed as a potential conflict of interest.
